# Two New Oleanane-Type Triterpenoids from Platycodi Radix and Anti-proliferative Activity in HSC-T6 Cells

**DOI:** 10.3390/molecules171214899

**Published:** 2012-12-13

**Authors:** Qin Zhan, Feng Zhang, Lianna Sun, Zhijun Wu, Wansheng Chen

**Affiliations:** 1 Department of Pharmacy, Changzheng Hospital, Second Military Medical University, 415 Fengyang Road, Shanghai 200003, China; E-Mails: zhanqin5@yahoo.com.cn (Q.Z.); fengzhangky@yahoo.com.cn (F.Z.); wuzhijun999@sina.com (Z.W.); 2 School of Pharmacy, Second Military Medical University, 325 Guohe Road, Shanghai 200433, China; E-Mail: sssnmr@yahoo.com.cn

**Keywords:** *Platycodon grandiflorum*, oleanane-type triterpenoid, triterpenoid saponin

## Abstract

Two new oleanane-type triterpenoids, named platycodonoids A and B (**1**, **2**), together with five known saponins, including platycodin D (**3**), deapioplatycodin D (**4**), 3-*O*-β-d-glucopyranosyl polygalacic acid (**5**), 3-*O*-β-d-glucopyranosyl platycodigenin (**6**) and polygalacin D (**7**), were isolated from the roots of *Platycodon grandiflorum*. On the basis of spectral data and chemical evidence, the structures of the new compounds were elucidated as 2β,3β,23,24-tetrahydroxy-28-nor-olean-12-en-16-one (**1**) and 2β,3β,23,24-tetrahydroxy-28-nor-olean-12-en-16-one-3-*O*-β-d-glucopyranoside (**2**). Compounds **1**–**7** were evaluated for their *in vitro* anti-proliferative activity against the HSC-T6 cell line.

## 1. Introduction

**Figure 1:**
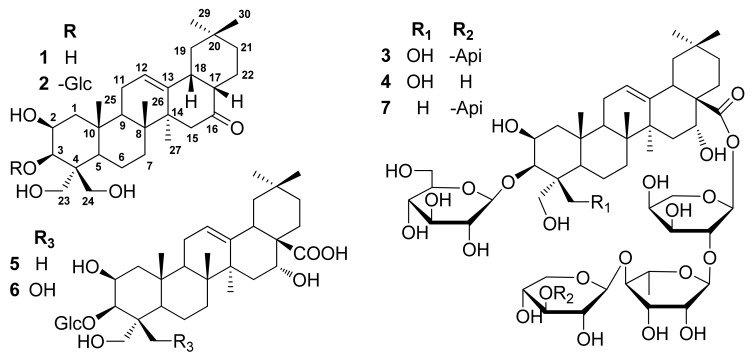
Structures of compounds **1**−**7**.

*Platycodon grandiflorum* A. DC. is a perennial plant in the family Campanulaceae which grows widely in East Asia. Platycodi Radix, the root of *P. grandiflorum*, has been used in traditional Oriental medicine as an expectorant for pulmonary disease and a remedy for respiratory disorders [1,2]. Platycodi Radix has recently been reported to exhibit many pharmacological activities, including anti-cancer properties [3,4,5,6,7,8], atopic dermatitis-like skin lesions treating [9,10,11], anti-skin photoaging effects [12], antiobesity and glucose metabolism regulation [13,14,15,16,17], anti-atherosclerotic [18], and anti-hyperlipidemic [19] activities. Chemical investigation of Platycodi Radix revealed that triterpenoid saponins were the main chemical components, and more than 55 triterpenoid saponins have been isolated from Platycodi Radix to date [20,21]. Based on the structures of the aglycones, the triterpenoid saponins are classified into three types: the platycodigenin type, platycogenic acid A lactone type and polygalacic acid type [22]. It has been previously demonstrated that Platycodi Radix showed protective effects against acute ethanol, acetaminophen-, carbon tetrachloride-, thioacetamide and cholestasis-induced hepatotoxicity or hepatic injury in mice and inhibited the progress of hepatic fibrosis in rats [23,24,25,26,27,28,29,30]. In our preliminary pharmacological study, the 70% EtOH extract of Platycodi Radix was also found to exhibit significant protective activities against liver fibrosis in rats. In a continued effort to search for possible hepatoprotective component from this herb, an investigation of Platycodi Radix was undertaken, and this has led to the isolation of two new triterpenoids [an aglycone and its saponin, named platycodonoids A (**1**) and B (**2**) (Figure 1)], which possess a rare 28-nor-oleanane-type with a C-16 keto group in the aglycone structure. Besides, five known saponins were isolated and identified as platycodin D (**3**) [31], deapio-platycodin D (**4**) [21], 3-*O*-β-d-glucopyranosyl polygalacic acid (**5**) [32], 3-*O*-β-d-glucopyranosyl platycodigenin (**6**) [33] and polygalacin D (**7**) [32,34], by comparison of their IR, NMR and MS data with literature values. To the best of our knowledge, it was first time triterpenoids such a platycodonoids A and B having a 28-nor-oleanane-type skeleton are reported from the genus *Platycodon*.

## 2. Results and Discussion

The air-dried roots of *P. Grandiflorum* were extracted three times with 70% EtOH under reflux. The combined extract was chromatographed over a macroporous adsorbing resin column and partitioned as described in the Experimental section. After repeated column chromatography, two new compounds **1** and **2** and five known saponins **3**–**7** were isolated and identified.

**Figure 2:**
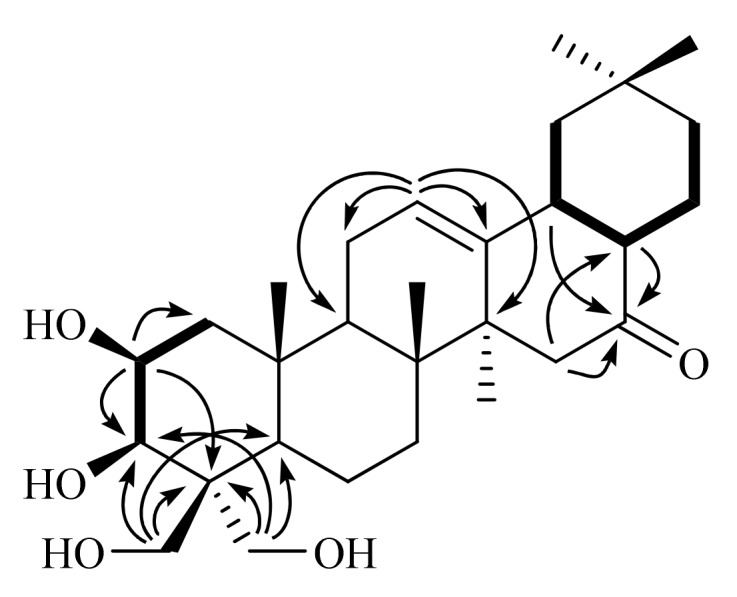
Key ^1^H-^1^H COSY (bold lines) and HMBC (H→C) correlations of compound **1**.

Compound **1** was obtained as white amorphous powder. Its molecular formula was assigned to be C_29_H_46_O_5_ based on the HRESIMS spectrum (*m/z* 497.3243 [M+Na]^+^, calcd. for C_29_H_46_O_5_Na, 497.3243). The IR spectrum exhibited absorptions at 3415, 2947, 1711, and 1385 cm^−1^ assignable to hydroxyl, methyl, ketone and methylene functions, respectively. The ^1^H-NMR spectrum of **1** showed the following signals: five tertiary methyl groups at *δ*_H_ 0.78, 0.84, 0.96, 1.14, and 1.63 (each 3H, s); two pairs of germinal oxygenated protons at *δ*_H_ 4.20, 4.21, 4.87, 5.20 (each, d, *J* = 10.8 Hz); two oxygenated protons at *δ*_H_ 4.39 (d, *J* = 3.6 Hz) and 4.58 (dt, *J* = 7.2, 3.6 Hz); and an olefinic proton at *δ*_H_ 5.38 (t, *J* = 3.6 Hz). The ^13^C-NMR spectrum revealed 29 carbon signals, which were further classified by DEPT and HSQC experiments as five methyls, ten methylenes (two oxygenated), six methines (two oxygenated), five quaternary carbons, one trisubstituted double bond (*δ*_C_ 123.3 and 142.9), and one keto carbonyl (*δ*_C_ 214.0) (Table 1). The aforementioned data implied that **1** was a nor-oleanane-type triterpenoid with four hydroxyls. In the HMBC spectrum, two pairs of germinal oxygenated protons at *δ*_H_ 4.20, 4.21, 4.87, 5.20 (H_2_-23, H_2_-24) showed correlations to C-3 (*δ*_C_ 75.2), C-4 (*δ*_C_ 48.1) and C-5 (*δ*_C_ 48.5), indicating that two hydroxyls were linked at C-23 and C-24. The two remaining hydroxyls were placed at C-2 (*δ*_C_ 72.0) and C-3, which were deduced by the HMBC correlations from the proton H-2 to C-1 (*δ*_C_ 44.7), C-3, and C-4, and from H-3 (*δ*_H_ 4.39) to C-1, C-2, C-3, C-4, C-23 (*δ*_C_ 64.1) and C-24 (*δ*_C_ 64.7) (Figure 2). The small coupling constant (*J*_2,3_ = 3.6 Hz) of H-2 and H-3 indicated the axial position of H-3 and the equatorial position of H-2, which was further confirmed by a NOESY correlation between H-2 and H-3. Moreover, the chemical shifts H_2_-15 at *δ*_H_ 2.55 (d, *J* = 14.4 Hz), 1.93 (d, *J* = 14.4 Hz) and C-15 at *δ*_C_ 47.2 were quite different from that reported for oleanane-type triterpenoid [platycoside O, H-15, *δ*_H_ 1.81 (dd, *J* = 15.0, 3.0 Hz), 2.55 (d, *J* = 12.0 Hz), C-15 (*δ*_C_ 36.1)] [21]. These results suggested the keto group (*δ*_C_ 214.0) was at C-16, which was confirmed by the HMBC spectrum correlations from H_2_-15, H-17 (*δ*_H_ 1.65), H-18 (*δ*_H_ 2.79) and H-22 (*δ*_H_ 1.34, 2.14) to C-16 (*δ*_C_ 214.0). The ^1^H-^1^H COSY spectrum of **1** indicated the presence of partial structures (Figure 2). Consequently, by comparison with the structure of platycoside O [21], compound **1** was determined to be 2β,3β,23,24-tetrahydroxy-28-nor-olean-12-en-16-one, and named platycodonoid A.

Compound **2** was obtained as white amorphous powder. It gave an [M+Na]^+^ peak at *m/z* 659.3728 in HRESIMS spectrum corresponding to the molecular formula of C_35_H_56_O_10_ (calcd. for C_35_H_56_O_10_Na, 659.3771), which showed a unit of C_6_H_10_O_5_ more than that of compound **1**. The 1D-NMR spectra of **2** displayed similarities to those of **1**, except for an additional sugar unit. An anomic proton signal at *δ*_H_ 5.15 (H-1', d, *J* = 7.8 Hz), an oxygenated methylene (H_2_-6') at *δ*_H_ 4.35 (t, *J* = 6.0 Hz) and 4.59 (d, *J* = 10.8 Hz), four oxymethine protons in the range *δ*_H_ 3.50−4.50 in the ^1^H-NMR spectrum suggested that **2** contained a sugar moiety. In accordance, the ^13^C-NMR spectrum displayed six carbon signals at *δ*_C_ 63.0, 72.0, 75.6, 79.0, 79.0, 106.6. The subsequent acid hydrolysis of **2** gave glucose only, which was analyzed by gas chromatography as glucitol acetate [35]. The linkage of this β-d-glucopyranose (*J* = 7.8 Hz) (Table 1) was confirmed by the HMBC correlation between H-1' and C-3. Therefore, the structure of platycodonoid B (**2**) was elucidated as 2β,3β,23,24-tetrahydroxy-28-nor-olean-12-en-16-one 3-*O*-β-d-glucopyranoside.

**Scheme 1:**
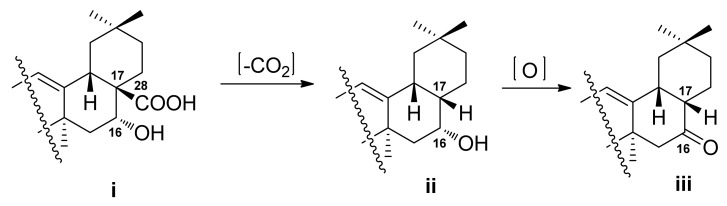
The plausible biogenetic origin of compounds **1** and **2**.

The origin of compounds **1** and **2** (Scheme 1) was proposed to be the oleanane-type triterpenoids **i** (compound **3**−**7**). Decarboxylation at C-28 would produce a key intermediate **ii**, which could undergo an oxidation at C-16 to yield **iii** (**1** and **2**).

Compounds **1**–**7** were evaluated for their anti-proliferative activities against the Hepatic Stellate Cell (HSC)-T6 line using the 3-(4,5-dimethylthiazol-2-yl)-2.5-diphenyltetrazolium bromide (MTT) assay [36]. Colchicine was used as positive control in this study (IC_50_ value < 10 μM). Among the tested compounds, compounds **1**, **3**, **4** and **7** were the most potent, showing IC_50_ values of 5.27, 1.77, 8.24 and 1.04 μM, respectively. The IC_50_ values for the remaing compounds, **2**, **5** and **6**, were 69.63, 8150.23, and 13.36 μM, respectively. It was reported that saponins from Platycodi Radix prevented the increase in the serum levels of hepatic enzyme markers (alanine aminotransferase and aspartate aminotransferase) and reduced oxidative stress, such as glutathione content and lipid peroxidation, in the liver in a dose-dependent manner [23,24,25,26,27,28,29,30]. The reason why compounds **1**, **3**, **4** and **7** delayed the formation of liver fibrosis need to be further studied. In the structure-activity relationship of these oleanane-type triterpenoids, the presence of a free carboxyl functional group at C-28 seemed not to be related to the hepatoprotective activity (compounds **5** and **6**). When forming C-28 glycosides, the presence of the glycosides affected the activity, and the number of monosaccharides in the sugar moiety increased the activity (compounds **3**, **4** and **7**). In contrast, when came to the 28-nor-oleanane-type triterpenoids **1** and **2**, the aglycone was more active than its corresponding glycoside.

## 3. Experimental

### 3.1. General

Optical rotations were measured with Perkin-Elmer 341 polarimeter. UV and IR spectra were recorded on Shimadzu UV-2550 and Perkin-Elmer 577 (using KBr disks) spectrophotometers, respectively. NMR spectra were acquired on a Bruker Avance III (600 MHz for ^1^H-NMR, ppm relative to TMS) spectrometer. ESIMS spectra were made on an Agilent 1200 series HPLC and interfaced to an Agilent 6410 triple-quadrupole mass spectrometer equipped with an electrospray ionization source, and HRESIMS spectra were made on an Agilent 1290 series HPLC and interfaced to an Agilent 6538 UHD Accurate-Mass Q-TOF LC/MS (Agilent Corporation, Wilmington, DE, USA). GC-MS was conducted on a Thermo Finnigan Trace GC apparatus using an L-Chirasil-Val column (25 m × 0.32 mm, i.d.). Semi-preparation RP-HPLC isolation was achieved with an Agilent 1200 instrument with refractive index detector (RID) using a YMC 5 μm C8 column (250 mm × 10 mm). Methanol for semi-preparative HPLC was of HPLC-grade (Merck, Darmstadt, Germany). Column chromatography: silica gel (200−300 mesh); macroporous adsorbing resin (D-101, ZTC-1, 0.3−1.2 mm, Tianjin Zhentiancheng Science & Technology Co., Ltd., Tianjin, China); sephadex LH-20 gel (40−70 μm, Amersham Pharmacia Biotech AB, Uppsala, Sweden); silica gel H (Qingdao Haiyang Chemical Co. Ltd., Qingdao, China). All solvents for column chromatography and acid hydrolysis were of analytical grade (Shanghai Chemical Reagents Company, Ltd., Shanghai, China). Spots of compounds on TLC were developed using 10% H_2_SO_4_-EtOH solution.

### 3.2. Plant Material

Platycodi Radix was collected from Taihe, Anhui Province, China, in September 2010 and was identified by Professor Hanming Zhang of School of Pharmacy, Second Military Medical University. A voucher specimen (No. 20100921) was deposited at the Department of Pharmacognosy, School of Pharmacy, Second Military Medical University.

### 3.3. Extraction and Isolution

Platycodi Radix was air-dried (10 kg) and extracted three times with 70% EtOH (50 L × 3 times) under reflux. The combined extract was concentrated *in vacuo* and suspended in water. The aqueous layer was chromatographed over a macroporous adsorbing resin column eluting with H_2_O, 30% EtOH, 60% EtOH and 95% EtOH. The 95% EtOH-eluted fraction (30 g) was applied to a silica gel column (CHCl_3_-MeOH, 50:1 to 10:1, v/v) and purified by semi-preparative HPLC (MeOH-H_2_O, 4:1) to give compound **1** (10.4 mg). The 60% EtOH-eluted fraction (120 g) was chromatographed on silica gel column eluting with a CHCl_3_-MeOH gradient (30:1 to 2:1, v/v) to afford five subfractions (A-E). Subfraction B (20 g) was chromatographed on a silica gel column (CHCl_3_-MeOH, 10:1 to 5:1, v/v), followed by Sephadex LH-20 column (MeOH-H_2_O, 1:1), and finally separated by semi-preparative HPLC (MeOH-H_2_O, 3:2) to yield compound **2** (9.3 mg). By the same procedure, compounds **3** (20.0 mg), **4** (8.7 mg), **5** (11.2 mg), **6** (9.4 mg) and **7** (7.6 mg) were obtained from subfractions C−E.

### 3.4. Characterization of Compound ***1*** and Compound ***2***

Compound **1**: white amorphous powder; ${\left[\alpha \right]}_{D}^{22.0}$ +44.3 (*c* 0.174, MeOH); IR (KBr) ν_max_ 3396, 2943, 2908, 1705, 1639, 1452, 1429, 1381, 1248, 1074, 1043, 898.7 cm^−1^; ^1^H-NMR and ^13^C-NMR data see Table 1; ESIMS *m/z* 497.4 [M+Na]^+^; HRESIMS *m/z* 497.3243 [M+Na]^+^ (calcd. for C_29_H_46_O_5_Na, 497.3243).

Compound **2**: white amorphous powder; ${\left[\alpha \right]}_{D}^{22.0}$ +59.9 (*c* 0.128, MeOH); IR (KBr) ν_max_ 3415, 2947, 1711, 1456, 1433, 1385, 1365, 1134, 1047, 696, 594 cm^−1^; ^1^H-NMR and ^13^C-NMR data see Table 1; ESIMS *m/z* 659.5 [M+Na]^+^; HRESIMS *m/z* 659.3728 [M+Na]^+^ (calcd. for C_35_H_56_O_10_Na, 659.3771).

### 3.5. Acid Hydrolysis of Compound ***2***

Compound **2** (3.0 mg) was refluxed with 1M HCl (dioxane-H_2_O, 1:1, 2 mL) at 90 °C for 3 h in a water bath. After dioxane was removed, the solution was extracted with EtOAc (2 mL × 3 times). After evaporating to dryness, the monosaccharide portion was analyzed by gas chromatography after conversion of the hydrolysates into corresponding alditol acetates. Only D-glucose was detected. The EtOAc portion was washed with H_2_O and evaporated to yield the aglycone. The aglycone was identified by TLC together with compound **1**.

### 3.6. *In Vitro* Inhibitory Activity on Cell Proliferation

Tested compounds **1**−**7** were dissolved in DMSO (final concertration, 0.1%). Inhibitory activity of compounds **1**−**7** against HSC-T6 cell line was evaluated by the MTT assay [36]. Briefly, cells at the exponential growth phase were harvested and seeded into a flatbottom 96-well plate. A total of 90 μL containing 5 × 10^4^ cells was added to each well of the plate and incubated for 24 h in a 5% humidified CO_2_ at 37 °C. HSC-T6 cells were treated with vehicle or compounds at concerntration of 0.01, 0.1, 1, 10, 100 and 1000 μg/mL. After 48 h of incubation at 37 °C, 20 μL/well, MTT was then added and the plate was again incubated at 37 °C for 4 h. Reduction of MTT to formazan was measured in an ELISA plate reader at 570 nm. Inhibitory activity of compounds **1**−**7** on cell proliferation (% of control) was calculated as 100 × (absorbance of treated compound—absorbance of background light)/(absorbance of control—absorbance of background light). Data were expressed as the mean of the three independent experiment. Colchicin was used as a positive control.

## 4. Conclusions

In conclusion, the phytochemical investigation of the roots extract of Platycodi Radix afforded two new triterpenoids, platycodonoids A (**1**) and B (**2**), together with five known triterpenoids: platycodin D (**3**), deapio platycodin D (**4**), 3-*O*-β-d-glucopyranosyl polygalacic acid (**5**), 3-*O*-β-d-glucopyranosyl platycodigenin (**6**) and polygalacin D (**7**). The structures of the compounds were elucidated on the basis of spectral analysis and chemical evidence and literature comparisons in the case of the known ones. Compounds **1**, **3**, **4** and **7** exhibited significant hepatoprotective activities against HSC-T6 cell lines *in vitro* (IC_50_ value < 10 μM).

## Figures and Tables

**Table 1:** ^1^H-NMR (600 MHz) and ^13^C-NMR (150 MHz) data for compounds **1** and **2** (pyridine-*d_5_*, *δ*_H_ in ppm, *J* in Hz).

Position	1		2	
	*δ*_C_	*δ*_H_	*δ*_C_	*δ*_H_
1	44.7	2.33 (1H, dd, 13.8, 3.0), 1.30 (1H, m)	45.0	2.08 (1H, m), 1.57 (1H, m)
2	72.0	4.58 (1H, dt, 7.2, 3.6)	70.0	4.76 (1H, m)
3	75.2	4.39 (1H, d, 3.6)	86.4	4.61 (1H, brs)
4	48.1		47.8	
5	48.5	1.82 (1H, brd, 12.0)	48.4	1.85 (1H, d, 12.0)
6	19.2	1.98 (1H, m), 1.83 (1H, brs)	19.4	1.92 (1H, m ), 1.61 (1H, m)
7	33.5	1.53 (1H, m), 0.91 (1H, m)	33.4	1.47 (1H, t, 13.8), 1.18 (1H, m)
8	40.1		40.3	
9	47.3	2.49 (1H, t, 5.4)	47.3	2.53 (1H, t, 5.4)
10	37.3		37.7	
11	24.2	2.05 (1H, m), 0.84(1H, m)	24.2	2.04 (1H, m), 0.88 (1H, m)
12	123.3	5.38 (1H, t, 3.6)	123.6	5.42 (1H, brs)
13	142.9		142.9	
14	46.9		47.1	
15	47.2	2.55 (1H, d, 14.4), 1.93 (1H, d, 14.4)	47.1	2.58 (1H, d, 14.4), 1.96 (1H, d, 14.4)
16	214.0		213.8	
17	48.1	1.65 (1H, m)	48.3	1.71 (1H, m)
18	45.1	2.79 (1H, m)	45.2	2.83 (1H, m)
19	47.0	1.40 (1H, t, 13.2), 1.17 (1H, m)	47.3	1.47 (1H, t, 13.2), 1.21(1H, m)
20	31.3		31.4	
21	35.0	1.09 (2H, m)	35.0	1.09 (2H, m)
22	21.5	2.14 (1H, m), 1.34 (1H, m)	21.6	2.17 (1H, m), 1.38 (1H, m)
23	64.1	4.87 (1H, d, 10.8), 4.20 (1H, d, 10.8)	63.8	4.99 (1H, d, 10.8), 4.11 (1H, d, 11.4)
24	64.7	5.20 (1H, d, 10.8), 4.21 (1H, d, 10.8)	63.8	4.80 (1H, d, 10.8), 4.26 (1H, d, 10.8)
25	17.9	0.96 (3H, s)	18.3	0.99 (3H, s)
26	17.6	1.63 (3H, s)	18.0	1.55 (3H, s)
27	27.3	1.14 (3H, s)	27.2	1.17 (3H, s)
29	33.6	0.78 (3H, s)	33.5	0.85 (3H, s)
30	23.7	0.84 (3H, s)	23.8	0.89 (3H, s)
1'			106.6	5.15 (1H, d, 7.8)
2'			75.6	4.05 (1H, t, 8.1)
3'			79.0	4.18 (1H, m)
4'			72.0	4.18 (1H, m)
5'			79.0	3.98 (1H, m)
6'			63.0	4.59 (1H, d, 10.8), 4.35 (1H, t, 6.0)
